# Leaf proteome modulation and cytological features of seagrass *Cymodocea nodosa* in response to long-term high CO_2_ exposure in volcanic vents

**DOI:** 10.1038/s41598-020-78764-7

**Published:** 2020-12-18

**Authors:** Amalia Piro, Letizia Bernardo, Ilia Anna Serra, Isabel Barrote, Irene Olivé, Monya M. Costa, Luigi Lucini, Rui Santos, Silvia Mazzuca, João Silva

**Affiliations:** 1 Laboratory of Plant Biology and Plant Proteomics (Lab.Bio.Pro.Ve), Department of Chemistry and Chemical Technologies, Università della Calabria, Ponte Bucci 12 C, 87036 Rende, CS Italy; 2 Department of Sustainable Food Process, Università Cattolica Sacro Cuore, Via Emilia Parmense 84, 29122 Piacenza, Italy; 3 CCMAR – Centre of Marine Sciences, University of Algarve, Campus of Gambelas, 8005-139 Faro, Portugal; 4 Departamento de Biología, Facultad de Ciencias del Mar Y Ambientales, Universidad de Cádiz, Cádiz, Spain

**Keywords:** Ecology, Molecular biology, Plant sciences

## Abstract

Seagrass *Cymodocea nodosa* was sampled off the Vulcano island, in the vicinity of a submarine volcanic vent. Leaf samples were collected from plants growing in a naturally acidified site, influenced by the long-term exposure to high CO_2_ emissions, and compared with others collected in a nearby meadow living at normal *p*CO_2_ conditions. The differential accumulated proteins in leaves growing in the two contrasting *p*CO_2_ environments was investigated. Acidified leaf tissues had less total protein content and the semi-quantitative proteomic comparison revealed a strong general depletion of proteins belonging to the carbon metabolism and protein metabolism. A very large accumulation of proteins related to the cell respiration and to light harvesting process was found in acidified leaves in comparison with those growing in the normal *p*CO_2_ site. The metabolic pathways linked to cytoskeleton turnover also seemed affected by the acidified condition, since a strong reduction in the concentration of cytoskeleton structural proteins was found in comparison with the normal *p*CO_2_ leaves. Results coming from the comparative proteomics were validated by the histological and cytological measurements, suggesting that the long lasting exposure and acclimation of *C. nodosa* to the vents involved phenotypic adjustments that can offer physiological and structural tools to survive the suboptimal conditions at the vents vicinity.

## Introduction

The Mediterranean submarine volcanic vents are natural sources of CO_2_ since this gas is the main component of the volcanic emissions that have been happening for hundreds of years, forming unique and extraordinary environments in which the relative abundance of dissolved inorganic carbon (Ci) species is altered by an increase in the partial pressure of CO_2_ (*p*CO_2_), with a consequent drastic reduction in seawater pH^1,2^. In these special acidic environments, marine ecosystems suffer from a drastic remodeling; while the pioneering studies on ocean acidification focused on how it negatively affects some species while favoring others^3^, more recent evidence exists on the large ecological effects on herbivores, invertebrates^4–7^ and on intra-community processes within seagrass meadows exposed to acidic conditions^8,9^. Seagrasses have been reported to be Ci-limited in the marine realm^10–12^, using CO_2_ and bicarbonate (HCO_3_^-^) as external Ci sources for photosynthesis^10^. Recent studies on ocean acidification have also aimed at resolving the question of whether seagrasses can fix an increasing amount of inorganic carbon (Ci) in the future, thus providing a way forward to their survival while alleviating the effects a more acidic seawater in their associated ecosystems (^12^ and references therein,^13–16^).

Volcanic vents create, in the present, the necessary acidified conditions to evaluate the long-lasting effect of high *p*CO_2_ exposure on acclimated populations of marine plants, which is a mandatory requirement to understand the plant’s real and sustained behavior^17^. Studies conducted in naturally acidified conditions at several volcanic sites have provided contrasting results, often suggesting species-specific responses to increased *p*CO_2_^8,9,18^. An ecological assessment of *Cymodocea nodosa* at a shallow acidified site at Volcano Island (Italy) revealed that the meadow is negatively affected by the environmental conditions at the low pH site, as the plant’s density and biomass decreased^8,9^; authors also reported a decrease in leaf area in plants acclimated to the CO_2_ vents. This latter finding, along with similar studies, strongly suggests that the acclimation of seagrasses to the long-lasting high *p*CO_2_ concentration encompasses several physiological and morphological adjustments. It is relevant to note that some biomechanical responses of *C. nodosa* were altered in the course of a CO_2_ enrichment experiment^19^ and that changes in plant anatomy and cell ultrastructure have been reported for *Halodule wrightii* under ocean acidification conditions^20^. These observations are in line to those previously observed in terrestrial plants, in which the exposure to high *p*CO_2_ induced several anatomical alterations^21–23^.

On the molecular side, a wider investigation on the gene expression profile, performed in the same population of *Cymodocea nodosa* in Vulcano island, confirmed the decrease in productivity in plants growing at the high CO_2_ site^24^. Contrastingly, the same study reported that productivity significantly increased with Ci availability in plants incubated with artificially CO_2_-enriched water at a non-acidified control site, supporting the hypothesis that *C. nodosa* might in general benefit from a higher Ci availability^24,25^. Taken all together, these results support the suggestion that volcanic vents may not be ideal analogues for ocean acidification studies and that the observed effects on seagrasses are not merely due to the increased CO_2_ availability but are also influenced by other environmental factors present at these sites^9^.

In this controversial scenario, our study aims to elucidate how a well-established natural population of *Cymodocea nodosa*, exposed to the CO_2_ vents environment at Vulcano Island, modulates its protein metabolism and what specific modifications take place, both at the morphological and functional traits levels, associated to the long-term adaptation process.

Comparative proteomics has been previously applied to seagrasses, revealing the protein molecular dynamics for surviving under various conditions^26–30^ Since the amounts of protein and transcripts corresponding to the same gene are generally loosely correlated^31^, the advantage offered by proteomics in the present study is to reveal changes in protein accumulation induced by high CO_2_ that cannot have been predicted from the previous transcriptomics investigation^24^, thus contributing to elucidate the effects of a long-term exposure to naturally increased *p*CO_2_.

## Results

### Protein yield, proteins identification and differential accumulated proteins in leaf tissues

**Figure 1:**
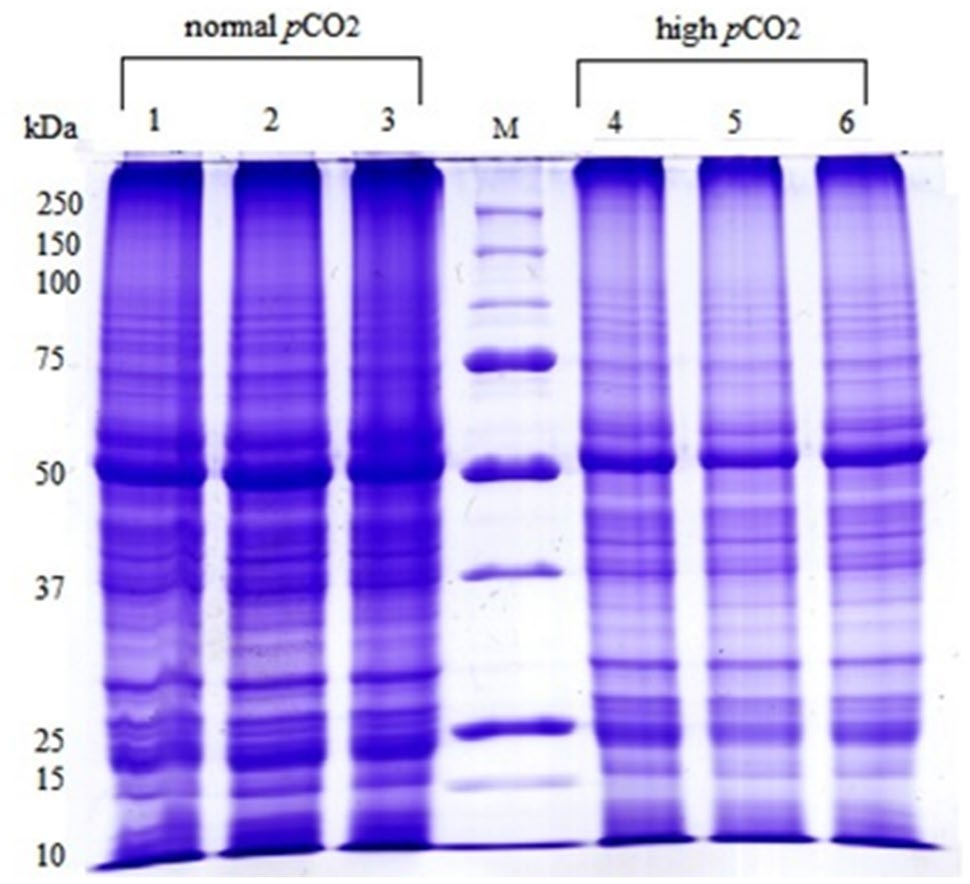
1D-SDS PAGE of proteins from leaves of three biological replicates of *Cymodocea nodosa* living in normal (lanes 1,2,3) and in high (lanes 4,5,6) *p*CO_2_ environments in Vulcano Island. 25 μg of proteins in each well were loaded. Markers used from Bio-Rad 250–10 kDa.

A decrease of 30% in protein yield in leaf tissues of plants growing in high *p*CO_2_ comparing to the normal *p*CO_2_ condition was found (See the Supplementary Table 1). The SDS-PAGEs of leaf proteins provided well-resolved lanes both in normal and high *p*CO_2_ samples. Each lane consists of about 80 different polypeptides bands, demonstrating the efficiency of the protein extraction and purification by means of the multistep protocol optimized for *C. nodosa*^28^. Spite the same amount of leaf proteins loaded on each well, the band at 55 kDa, corresponding to the large subunit of RuBisCo, decreased in all replicates of plants living in high *p*CO_2_ with respect to those under normal *p*CO_2_ condition (Fig. 1). Measures from the digitalized images of the gels by the Quantity One 1-D Analysis Software (Bio-Rad Laboratories; Berkley, California) gave a mean decrease of up to 40% in the optical density of the 55 kDa band (data not shown) in the high *p*CO_2_ samples.

**Figure 2:**
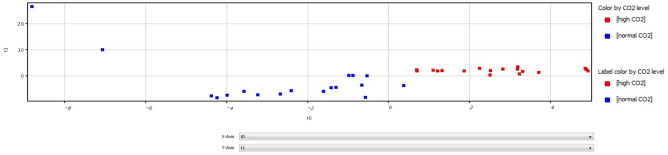
Multivariate classification (PLS-DA) predictions (Mass Profiler Professional Software) of full mass spectra results from normal *p*CO_2_ (blue box) and high *p*CO_2_ (red box) *C. nodosa* samples (All mass spectra of all samples). Horizontal dashed lines indicate the thresholds and vertical dashed lines indicate the separation between samples.

Figure 2 depicts the multivariate classification (PLS-DA) of all mass spectra results from normal *p*CO_2_ and high *p*CO_2_ plants. Spectra patterns of normal (blue dots) and high (red dots) *p*CO_2_ plants are quite distinct. Plants collected in high *p*CO_2_ showed a higher degree of homogeneity in comparison to samples collected in normal *p*CO_2_.

**Figure 3:**
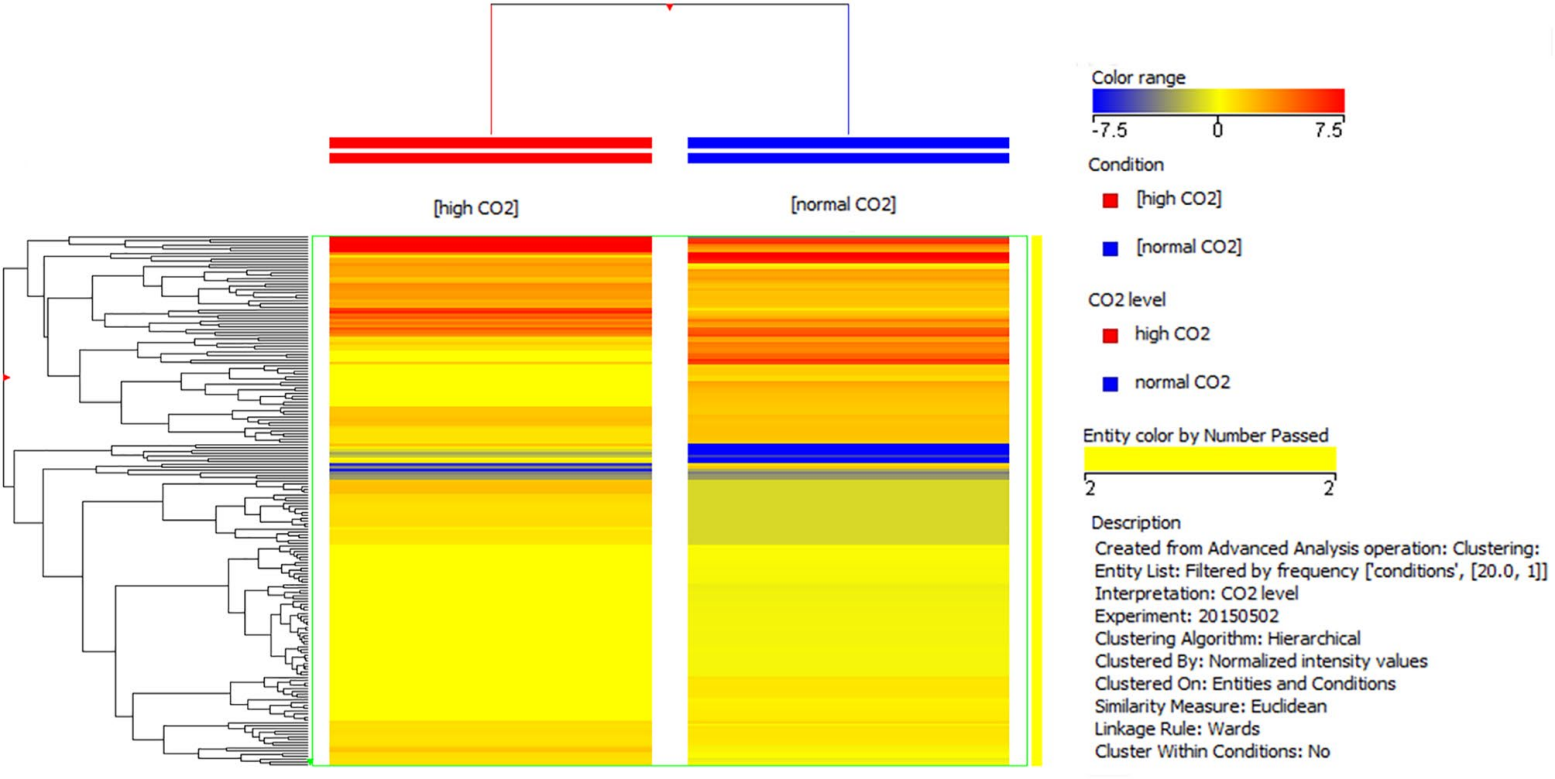
Hierarchical clustering of all proteins identified in the normal *p*CO_2_ and in the high *p*CO_2_ samples.

The mass spectra analysis coupled with database search has identified 190 proteins in all samples (Supplementary Tables 2 and 3). The hierarchical clustering of all identified proteins is shown in Fig. 3. Under the screening criteria of a fold change greater than 2 or less than 0.60 and *p* value < 0.05, a total of 75 proteins were identified to be differentially abundant (DAPs) by comparison between normal *p*CO_2_ and high *p*CO_2_ plants; 45 proteins resulted accumulated, while the remaining 30 proteins are depleted. All these proteins were regarded as candidate proteins associated with the high *p*CO_2_ adaptation and acclimation processes (Table 1).

**Table 1:** Differential abundant proteins (DAPs) in leaf tissue of high *p*CO2 samples comparing with those of normal *p*CO2 samples. Accession number, protein name, fold change expressed as Log (2) and absorbance, protein behavior KEGG orthology, molecular mass and metabolisms have been shown. Strongly accumulated and strongly depleted proteins are reported in bold. Details on mass spectrometry parameters for peptides for each identified proteins are reported in the Supplementary Table 2

Swiss-Prot ID	KEGG* orthology (proteins)	Protein	Log FC ([high CO2] versus [normal CO2]) Normalized	FC (abs) ([high CO2] versus [normal CO2]) Normalized	DAPs ([high CO2] versus [normal CO2]) Normalized	Molecular Mass (Da)	KEGG* orthology (metabolisms)	Metabolism
P0C365	K02705	Photosystem II CP43 chlorophyll apoprotein	13.134	8993.973	STA	52,246.3	ko00195	Photosynthesis
P0C435	K02706	Photosystem II D2 protein	12.430	5520.685	STA	39,801.0	ko00195	Photosynthesis
P05641	K02704	Photosystem II CP47 chlorophyll apoprotein	10.513	1461.336	STA	56,276.2	ko00195	Photosynthesis
P0C432	K02703	Photosystem Q(B) protein	8.324	320.653	STA	39,076.0	ko00195	Photosynthesis
P05642	K02635	Cytochrome b6	7.938	245.255	STA	24,310.3	ko00195	Photosynthesis
P19023	K02133	ATP synthase subunit beta, mitochondrial	7.155	142.595	STA	59,216.4	ko00190	Energy metabolism
P00827	K02112	ATP synthase subunit beta, chloroplastic	6.426	86.00222	STA	54,097.0	ko00195	Photosynthesis
P0C387	K02634	Apocytochrome f	6.121	69.602	STA	35,580.5	ko00195	Photosynthesis
A5H454	K00432	Peroxidase 66	5.701	52.048	STA	33,932.3	ko01100	Lipid metabolism
P0C356	K02690	Photosystem I P700 chlorophyll a apoprotein A2	4.901	29.885	STA	82,672.8	ko00195	Photosynthesis
P12863	K01803	Triosephosphate isomerase, cytosolic	4.827	28.386	STA	27,252.6	ko00010	Glycolysis/Gluconeogenesis
O24592	K09840	9-cis-epoxycarotenoid dioxygenase 1, chloroplastic	4.805	27.953	STA	66,007.5	ko01110	Biosynthesis of seconday metabolites
P0C2Z4	K02111	ATP synthase subunit alpha, chloroplastic	4.095	17.091	STA	55,721.0	ko00195	Photosynthesis
P0C353	K02689	Photosystem I P700 chlorophyll a apoprotein A1	3.406	10.603	STA	83,395.2	ko00195	Photosynthesis
Q41764	K10363	Actin-depolymerizing factor 3	2.982	7.905	A	16,013.8	ko04812	Signaling and cellular processes
Q8W2B7	K13227	DIMBOA UDP-glucosyltransferase BX8	2.981	7.895	A	49,926.0	ko00402	Biosynthesis of seconday metabolites
P46302	K02979	40S ribosomal protein S28	2.980	7.889	A	7,467.6	ko03010	Translation
A1Y2B7	no KO assigned	Protein SUPPRESSOR OF GENE SILENCING 3	2.933	7.640	A	67,979.5	no KO assigned	No assigned metabolism
P46252	K02943	60S acidic ribosomal protein P2A	2.763	6.790	A	11,476.7	ko03010	Translation
Q00827	K08912	Chlorophyll a-b binding protein 48, chloroplastic	2.675	6.386	A	28,299.8	ko00195	Photosynthesis
A5H452	K00432	Peroxidase 70	2.449	5.462	A	33,994.0	ko01100	Lipid metabolism
Q9FQA3	K00799	Glutathione transferase GST 23	2.327	5.018	A	24,992.4	ko00480	Glutathione metabolism
B4FGS2	no KO assigned	Spindle and kinetochore-associated protein 1	2.254	4.769	A	30,488.3	no KO assigned	No assigned metabolism
B6TZD1	K08963	Methylthioribose-1-phosphate isomerase	2.205	4.613	A	38,735.5	ko00270	Amino acid metabolism
P46420	K00799	Glutathione S-transferase 4	2.178	4.524	A	24,741.1	ko00480	Glutathione metabolism
P11155	K20115	Pyruvate, phosphate dikinase 1, chloroplastic	2.143	4.416	A	103,585.5	ko00710	Carbon fixation
P49101	K06103	Calcium-dependent protein kinase 2	2.139	4.405	A	58,422.9	ko04131	Exocytosis
B8A031	K03644	Lipoyl synthase, mitochondrial	2.126	4.366	A	42,341.6	ko01100	Lipid metabolism
P49094	K01953	Asparagine synthetase [glutamine-hydrolyzing]	2.126	4.364	A	67,147.1	ko00270	Amino acid metabolism
P0C8M8	K08852	serine/threonine-protein kinase CCRP1	2.062	4.176	A	70,746.2	ko04141	Protein processing in endoplasmic reticulum
O63066	K10956	Preprotein translocase subunit SECY, chloroplastic	2.031	4.086	A	59,637.9	ko04141	Protein processing in endoplasmic reticulum
Q8LPU4	K11303	Histone acetyltransferase type B catalytic subunit	2.028	4.079	A	53,119.5	ko03400	DNA repair
C0PF72	K00620	Arginine biosynthesis bifunctional protein ArgJ, chloroplastic	2.016	4.046	A	48,407.3	ko01230	Amino acids biosynthesis
Q67EU8	K04482	DNA repair protein RAD51 homolog A	1.998	3.994	SLA	36,989.5	ko03400	DNA repair
Q10717	K16290	Cysteine proteinase 2	1.976	3.935	SLA	39,712.1	ko01002	Protein degradation
P41978	K04564	Superoxide dismutase [Mn] 3.2, mitochondrial	1.914	3.770	SLA	25,356.4	ko04146	Oxidative stress
P42390	K13222	Indole-3-glycerol phosphate lyase, chloroplastic	1.888	3.701	SLA	36,691.8	ko00402	Biosynthesis seconday metabolites
P00056	K00413	Cytochrome c	1.885	3.695	SLA	12,132.6	ko00190	Energy metabolism
P49081	K01638	Malate synthase, glyoxysomal	1.881	3.684	SLA	62,092.2	ko01200	Carbon metabolism
Q9XGD5	K00588	Caffeoyl-CoA O-methyltransferase 2	1.851	3.607	SLA	29,522.0	ko01110	Biosynthesis of secondary metabolites
P12959	K21632	Regulatory protein opaque-2	1.815	3.519	SLA	49,812.2	ko03000	Transcription
P23345	K04565	Superoxide dismutase [Cu-Zn] 4A	1.593	3.017	SLA	15,228.5	ko04146	Oxidativ stress
Q05737	K07874	GTP-binding protein YPTM2	1.558	2.945	SLA	22,646.2	ko04031	Protein transport
P06671	K08913	Chlorophyll a-b binding protein, chloroplastic	1.427	2.689	SLA	28,165.7	ko00195	Photosynthesis
P0C520	K02132	ATP synthase subunit alpha, mitochondrial	1.414	2.665	SLA	55,657.7	ko00190	Energy metabolism
Q41803	K03231	Elongation factor 1-alpha	−7.805	223.659	STD	49,574.4	ko03013	Translation
Q08062	K00025	Malate dehydrogenase, cytoplasmic	−7.712	209.621	STD	35,931.6	ko01200	Carbon metabolism
P0C510	K01601	Ribulose bisphosphate carboxylase large chain	−7.099	137.062	STD	53,450.7	ko00710	Carbon fixation
Q43298	K04077	Chaperonin CPN60-2, mitochondrial	−6.089	68.074	STD	61,219.3	ko03018	Protein folding
P27923	K02977	Ubiquitin-40S ribosomal protein S27a	−5.271	38.628	STD	17,909.5	hsa03010	Translation
P14640	K07374	Tubulin alpha-1 chain	−4.601	24.271	STD	50,414.8	ko04514	Citoskeleton metabolism
Q02245	K07374	Tubulin alpha-5 chain	−4.556	23.538	STD	50,251.7	ko04514	Citoskeleton metabolism
Q9ZT00	K19199	Ribulose bisphosphate carboxylase/oxygenase activase, chloroplastic	−4.483	22.367	STD	48,108.8	ko00710	Carbon fixation
P09315	K05298	Glyceraldehyde-3-phosphate dehydrogenase A, chloroplastic	−4.386	20.907	STD	43,208.4	ko00010	Glycolysis/Gluconeogenesis
Q7SIC9	K00615	Transketolase, chloroplastic	−4.094	17.078	STD	73,391.4	ko01200	Carbon metabolism
P24631	K13993	17.5 kDa class II heat shock protein	−3.6320088	12.398	STD	17,568.0	ko04141	Protein processing in endoplasmic reticulum
P08440	K01623	Fructose-bisphosphate aldolase, cytoplasmic	−3.430	10.778	STD	39,059.9	ko00010	Glycolysis/Gluconeogenesis
P26301	K01689	Enolase 1	−2.870	7.313	D	48,290.9	ko00010	Glycolysis/Gluconeogenesis
P04712	K00695	Sucrose synthase 1	−2.545	5.83469	D	92,129.6	ko00500	Starch and sucrose metabolism
Q43704	K02541	DNA replication licensing factor MCM3	−2.539	5.811	D	85,694.0	ko03030	DNA replication and repair
P15719	K00051	Malate dehydrogenase [NADP], chloroplastic	−2.475	5.560	D	47,429.3	ko01200	Carbon metabolism
P0C1M0	K02115	ATP synthase subunit gamma, chloroplastic	−2.464	5.516	D	40,131.4	ko00195	Photosynthesis
P38560	K01915	Glutamine synthetase root isozyme 2	−2.115	4.332	D	40,492.8	ko00250	Amino acid Biosynthesis
P18122	K03781	Catalase isozyme 1	−2.073	4.207	D	57,389.9	ko04146	Oxidativ stress
P02582	K06759	Actin-1	−2.058	4.164	D	41,902.1	ko04514	Citoskeleton metabolism
Q6XZ79	K00847	Fructokinase-1	−2.028	4.078	D	34,861.4	ko00500	Starch and sucrose metabolism
Q9SP22	K08057	Calreticulin	−1.854	3.616	SLD	48,052.8	ko04141	Protein folding and sorting
O22424	K02987	40S ribosomal protein S4	−1.832	3.561	SLD	30,130.6	ko03013	Translation
Q195N6	K01006	Pyruvate, phosphate dikinase regulatory protein, chloroplastic	−1.804	3.491	SLD	46,360.8	ko00710	Carbon fixation
B4G072	K13227	DIMBOA UDP-glucosyltransferase BX9	−1.710	3.272	SLD	50,358.7	ko00402	Biosynthesis seconday metabolites
Q9ZSV1	K24070	Poly [ADP-ribose] polymerase 1	−1.698	3.244	SLD	111,614.5	ko03410	Dna repair
P80607	K13379	Alpha-1,4-glucan-protein synthase [UDP-forming]	−1.654	3.148	SLD	41,717.1	ko00520	Carbohydrate metabolism
B4FAT0	K11996	Adenylyltransferase and sulfurtransferase MOCS3 2	−1.514	2.856	SLD	52,564.9	ko03013	Translation
Q8S4P4	K11430	Histone-lysine N-methyltransferase EZ3	−1.498	2.824	SLD	102,388.1	ko00270	Amino acid metabolism
Q43272	K00131	NADP-dependent glyceraldehyde-3-phosphate dehydrogenase	−1.432	2.698	SLD	53,773.0	ko00010	Glycolysis/Gluconeogenesis

STA: Strongly accumulated; STD: Strongly depleted; A: accumulated; D: Depleted; SLA: Slightly accumulated; SLD: Slightly depleted

* KEGG codes are developed in the Kanehisa Laboratories

**Figure 4:**
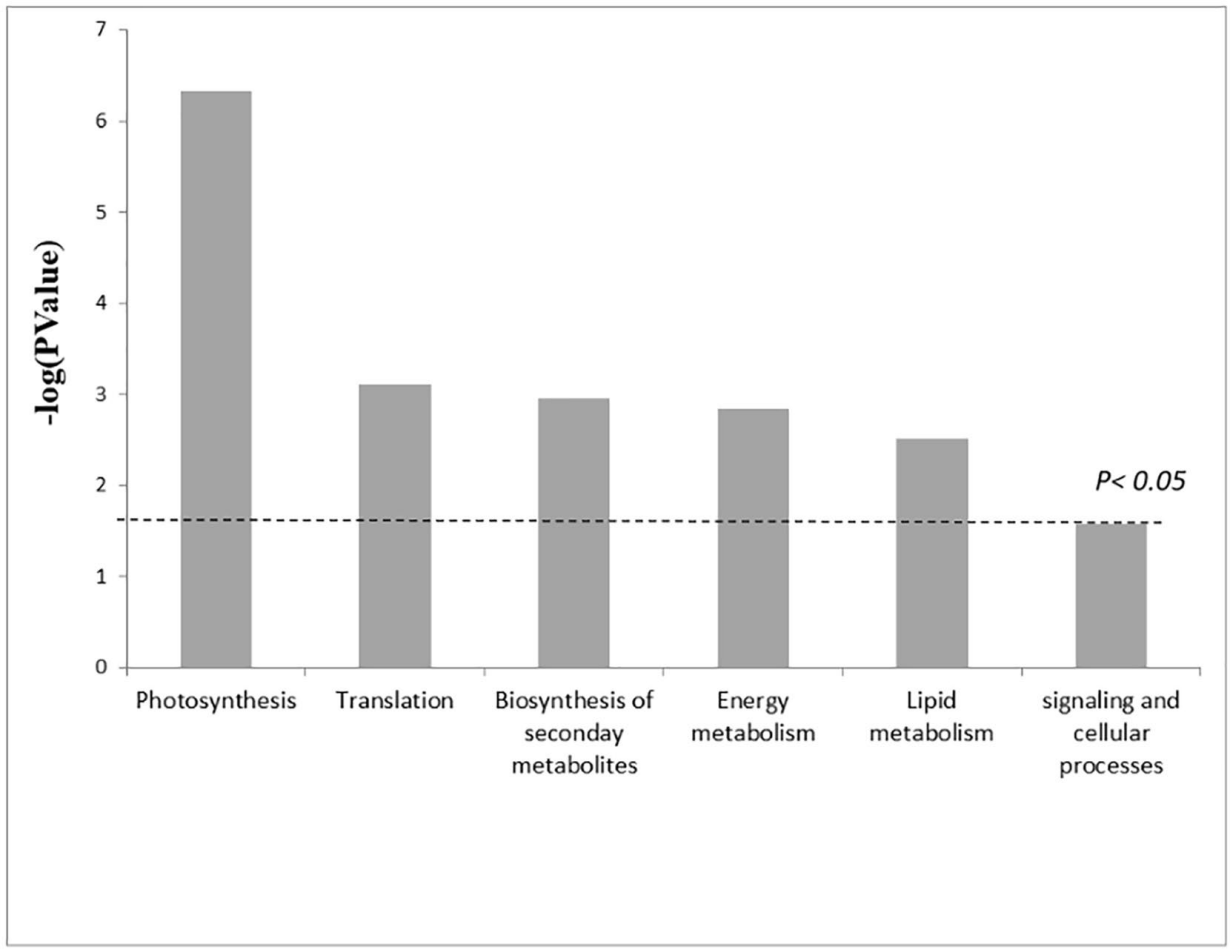
KEGG pathways where the differentially abundant proteins were enriched. The x-axis shows the proteins involved in the extended KEGG network and pathways. P values were calculated using a modified Fisher’s exact test. Values above the threshold indicate p < 0.05. KEGG pathways are developed by Kanehisa Laboratories^32^.

The pathway analysis using the Kyoto Encyclopedia of Genes and Genomes (KEGG) pathway database^32^ (http://www.genome.jp/kegg/pathway.html, accessed on 11 September 2019) identified 6 pathways (p < 0.05) related to proteins with enriched relative abundance, as shown in Fig. 4. Proteins involved in the light reactions of photosynthesis are the most relevantly enriched under the acidified conditions. These include the Photosystem II CP43 chlorophyll apoprotein, Photosystem II D2 protein, Photosystem II CP47 chlorophyll apoprotein, Photosystem Q(B) protein, Photosystem I P700 chlorophyll a apoprotein A2, ATP synthase subunit beta, chloroplastic, Cytochrome b6, ATP synthase subunit alpha and the chloroplastic Photosystem I P700 chlorophyll a apoprotein A1 (Table 1). In contrast, the depleted metabolic pathways were those related to carbon fixation, carbon metabolism, glycolysis/gluconeogenesis. The Ribulose bisphosphate carboxylase large chain and the Ribulose bisphosphate carboxylase/oxygenase activase appeared strongly depleted in acidified conditions, as also cytoplasmic and chloroplastic Malate dehydrogenase and Transketolase. The key enzymes of glycolysis Glyceraldehyde-3-phosphate dehydrogenase A, Fructose-bisphosphate aldolase and Enolase 1 were also depleted. The Malate synthase, that facilitates the glyoxylate cycle, the Pyruvate phosphate dikinase involved in the alternative glycolisis,, the Serine-threonine protein and many proteins involved in the amino acid metabolism are enriched under acidified conditions. Also the glutathione metabolism seems to be upregulated as the Glutathione transferases are accumulated under acidified condition.

Protein folding and turnover seemed also to be affected under acidified conditions as the Elongation factor 1-alpha, Chaperonin CPN60-2 and Ubiquitin-40S ribosomal protein S27a were strongly depleted. Proteins belonging to the cytoskeleton metabolism are affected by acidification such as Tubulin alpha-1 chain, Tubulin alpha-5 chain and Actin-1 (Table 1).

**Figure 5:**
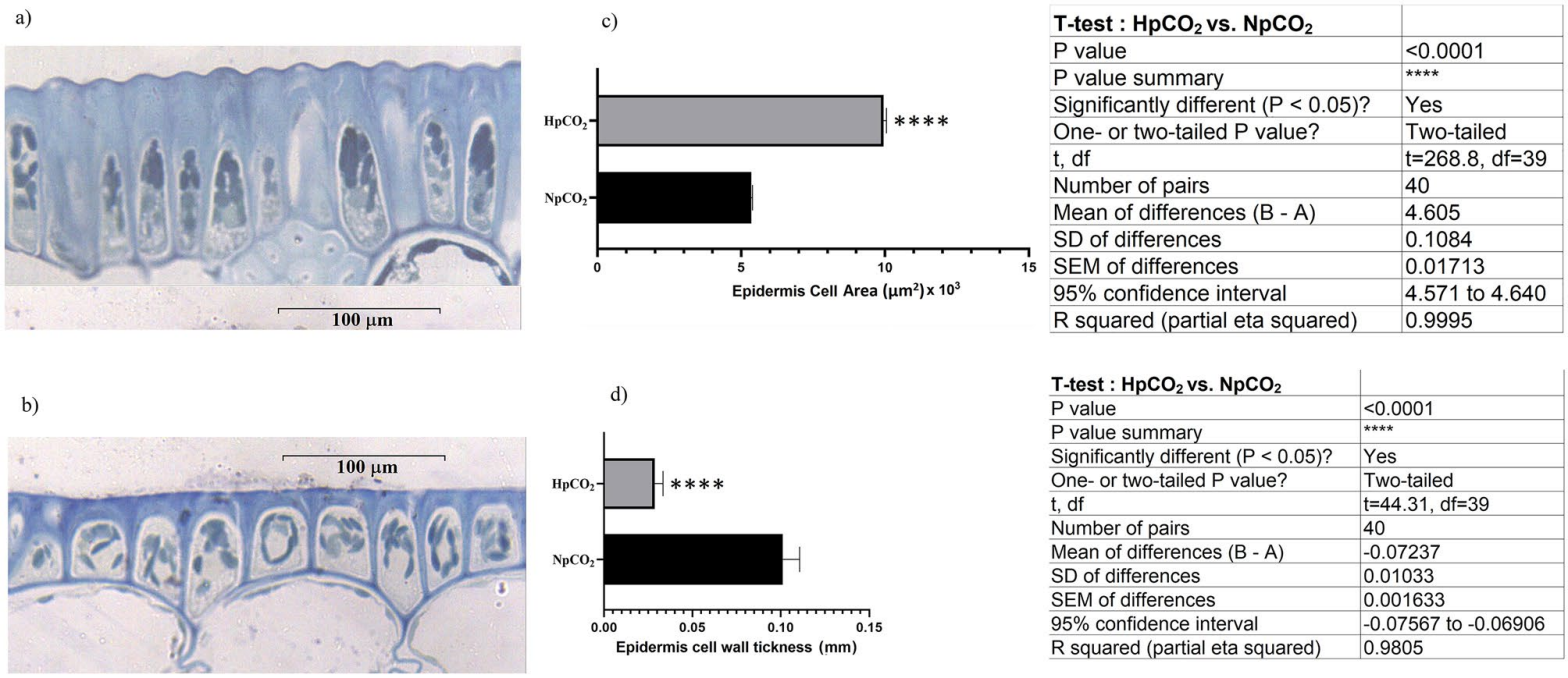
Cytological measurements of leaf epidermis of *Cymodocea nodosa* plants growing in normal and high *p*CO_2_ environments. Epidermal leaf cell microphotographs of *C. nodosa* growing in normal (**a**) and high (**b**) *p*CO_2_ environments. Boxplots (± SD) showing the cell area (**c**) and cell wall thickness of epidermal thickness (**d**) of *C. nodosa* growing in normal and high *p*CO_2_ environments.

The leaf blades of *C. nodosa* growing under acidified conditions were shown to be almost 15% wider (3.22 ± 0.43 mm) than those of plants living in normal *p*CO_2_ (2.76 ± 0.52 mm); epidermal cells have larger areas and thinner cell walls in high *p*CO_2_ leaves than those of normal *p*CO_2_ cells (Fig. 5). Leaves growing under acidified conditions have also larger parenchyma cells, lesser number of cells/mm^2^ and thinner cell wall than those of normal *p*CO_2_ cells (Supplementary Table 4).

**Figure 6:**
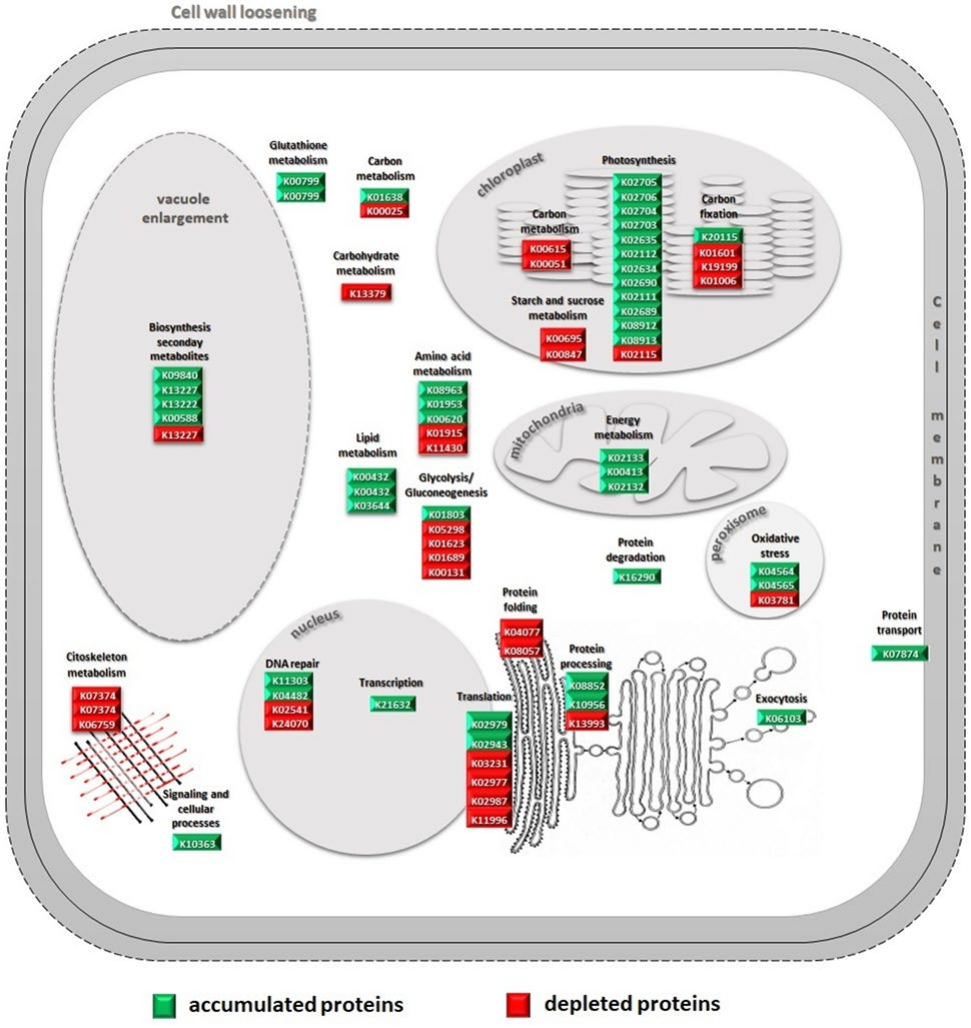
Schematic diagram of differentially expressed proteins belonging to metabolic pathways/cellular processes leading to the acclimation/tolerance of *Cymodocea nodosa* in volcanic vents. The acclimation strategy combines the reduction of carbon fixation, gluconeogenesis, carbohydrate metabolism and protein synthesis with increasing photophosphorylation, cell respiration and aminoacid metabolism to maintain the high energy demand for leaf expansion and elongation of the mesophyll cell; the cell expansion is accomplished by the cell wall loosening, the vacuole enlargement and the cytoskeleton remodeling. Proteins belonging the oxidative stress response pathway, the Gluthatione metabolism and the biosynthesis of secondary metabolites were also accumulated, suggesting that potential external stress factor other than CO_2_ are at play at the Vulcano submarine vents. Proteins and related KEGG codes, reported also in Table 1, are developed by Kanehisa Laboratories^32^.

Schematic representation of DAPs involved in different metabolic pathways/cellular processes in *Cymodocea nodosa* to cope the environmental conditions at CO_2_ vents is reported in the Fig. 6

.

## Discussion

The comparative proteomics data showed that the long-term exposure to high *p*CO_2_ in the vicinity of volcanic vents strongly affected the inorganic carbon assimilation in leaves of *Cymodocea nodosa* as demonstrated by the significantly decreased levels of the key carbon metabolism enzymes . These results suggest that the chronic exposure of *C. nodosa* to CO_2_-enriched volcanic emissions did not act positively toward carbon fixation, neither via Rubisco nor via PEPC, indicating a general depression of both inorganic carbon fixation pathways; even if the accumulation of Malate synthase and Pyruvate dikinase can pose the question whether or not seagrasses have a carbon concentrating mechanism, its existence to date is not proven and more evidence from “omics” is still required.

Our proteomic findings reinforce the results of a gene expression study carried out simultaneously on plants from the same populations, in which a significant down-regulation of the transcripts related to carbon metabolism, carbon uptake and carbohydrate metabolism were also found to be strongly down-regulated in the acidified leaves^25^. Proteomics and trascriptomics thus demonstrate that the long-lasting exposure to the vents conditions lead to the overall depression of the primary metabolisms, and are the probable cause of the reduction of the net plant productivity (NPP) and plant biomass found in plants growing in the vicinity of the vents^8,25^.

The metabolism of proteins is also negatively affected; previous proteomics studies of seagrasses living under several acute stressors or long-lasting disturbing factors also reported lower protein content linked to depletion of Rubisco^28–30^. Here we found that the lower protein contents is mainly due to an impaired protein synthesis at the post-transcriptional level in leaf tissue of plants under acidified conditions. Also protein function and turnover seems be the machinery that require an increasing energy demand.

Despite the strong depletion of the Calvin cycle proteins, a significant positive correlation between proteins related to the light reactions of photosynthesis and the exposure to high *p*CO_2_ was found, the same happening with some proteins belonging to photosystem II and photosystem I. High levels of Chl *a* and significant increases in maximum electron transport rate and in compensation irradiance were previously found in *C. nodosa* grown under acidified conditions at this same volcanic CO_2_ vent, corroborating the hypothesis that acidification promotes the photosynthetic light reactions^8^. Moreover, we found that the energy metabolism from photophosphorylation and from oxidative phosphorylation are positively affected by high *p*CO_2_ exposure; the latter comes from leaf mitochondrial respiration which have been previously found to be up-regulated in the response to high *p*CO2^24,33^. The observed depletion of the Calvin cycle proteins near the vents is likely to reflect a higher efficiency in the use of CO_2_ that would require a higher energy availability from the photosynthetic electron transport chain. On the other hand, a potential imbalance between the photosynthetic electron transport chain and the Calvin cycle reactions could also result in the formation of reactive oxygen species (ROS).

Increases in ROS may result from a number of other stress factors, and are usually associated with increases in the overall antioxidant capacity. The observed accumulation of antioxidant enzymes such as Peroxidases, Glutathione S-transferases, Superoxide dismutases and enzymes belonging the phenylpropanoid pathway has been well documented as defense responses of seagrasses to light stress and heavy metal toxicity^27,29^. The enhanced multi-enzyme antioxidant system indicates that vents conditions results in ROS production, triggering the response to scavenge H_2_O_2_ and maintaining the cell redox status. Taken all together, our results support the previously conveyed idea that potential external stress factors other than CO_2_ are at play at the Vulcano submarine vents, significantly affecting the plants’ metabolic balance^8,9,34^.

The adaptive strategy that plants use to cope with vents condition also involves some morphological adjustments. If at the meadow level, *C. nodosa* lowered the density, biomass and below/aboveground biomass ratio at the acidified site^8,9^, at plant level, no significant differences in number of leaves per shoot (total mean number of leaves per shoot was 4 ± 0.7, data not shown) was found. Interestingly, we found that plants growing closer to the vents had shorter but wider leaf blades, epidermal cells with thinner cell walls and larger parenchyma cells. These morphological differences could indicate ecotypes, eventually selected by this extreme environment, but results from the population genetics carried out on the same sampling site showed no genetic differentiation and a high gene flow between *C. nodosa* plants growing at both the acidified and the control sites^25^. The morphological differences found should then be considered as a phenotypical response of *C. nodosa* to the pressure of the acidified environment. A similar pattern of leaf parameters was also recently described at the Vulcano CO_2_ vents by Vizzini et al.^9^. Our data support the hypothesis that under acidified conditions the cell expansion contributed more than cell division to the leaf expansion; following this assumption, leaf blades become shorter and wider than those grown in normal *p*CO_2_ condition. Further studies conducted on *C. nodosa* and *Halodule wrightii* demonstrated that an elevated CO_2_ concentration has effects on leaf mechanical resistence such as on the leaf anatomy and cell ultrastructures^19,20^. The authors reported that high *p*CO_2_-grown *C. nodosa* had an increased leaf-breaking force related to leaf growth; leaf width and cross-section area were larger under acidification in *Halodule wrigtii*, thus indicating that increased CO_2_ may manifest in large part at cellular level. Here we might conclude that the morphological traits have shown a positive correlation between mesophyll cell size and pH at CO_2_ venting sites, suggesting that wider leaves have a higher capacity to buffer pH. Even if is demonstrated that exposure to elevate pCO_2_ alters plant structure by inducing change in rate of cell division and cell expansion in seed plants^35^, further investigation needs to elucidate whether *C. nodosa* growing in the vicinity of volcanic vents might ameliorate potential adverse effects on growth by means the mesophyll cell expansion and modified cell water uptake. In support of this idea, Ruocco et al.^24^ found that *C. nodosa* exposed to high *p*CO_2_ overexpressed transcripts encoding for enzymes that play an integral role in pH homoeostasis of the cells. Under this concept, mesophyll cells of *C. nodosa* might couple the ions homeostasis with increased water uptake to adjust the osmotic balance.

Moreover, specific molecular rearrangements seem to validate the hypothesis that high *p*CO_2_ led to larger cell size; biosynthesis of secondary metabolites appeared to be positively related to acidification and also to hormone-mediated response such ABA biosynthesis^36–38^. The actin-depolymerizing Factor 3 coupled with the Calcium-dependent protein kinase 2 (CDPK) have been found to modulate the plant cell shape through the regulation of the actin filament network in cytoskeleton^39^ and also to have a role in the re-organization of plant cytoplasm in response to a wide range of internal and external stimuli, suggesting a direct correlation between signal transduction and actin cytoskeleton reorganization in plants^40,41^. The strong depletion of tubulin and actin cytoskeleton constituents further support the suggestion that acidification affects the cytoskeleton dynamics and might trigger the modulation of cell enlargement and elongation in mesophyll cells. A pattern of thinner cell walls was also found in mesophyll epidermal cells of plants growing in the acidified site. Seagrasses possess a very different cell wall composition as well as proportion of polysaccharide and monosaccharides than terrestrial plants^42^; the modified cell wall structure and metabolism lead to an increase in the polyanionic character of seagrass cell wall^43^. It is well known that an acid cell wall is necessary for wall loosening to occur, thus promoting cell expansion and growth^44^; this mechanism is induced by the hormone auxin during cell elongation. In seagrasses, under normal conditions, the extracellular carbonic anhydrase mediates the conversion of HCO^3–^ to CO_2_ generating acid zones created by H^+^ extrusion from the cytoplasm to the cell wall^11^; we can speculate that in an acidified environment the increased exogenous protons could, at least partially, substitute auxin in inducing cell enlargement. The thinner cell wall found in acidified epidermal cells of *C. nodosa* is likely to come from the cell elongation without *ex novo* biosynthesis of structural wall carbohydrates, due to the impaired primary metabolism and depressed carbon fixation; the lowered biomass of plants exposed to high *p*CO_2_ also indicates that leaf elongation occurred mainly by means of cell expansion. Thus, cell wall seems to be a critical player in response to acidification and further studies on the cell wall metabolism of *C. nodosa* growing near Vulcano CO_2_ vents are necessary.

In conclusion, proteomic analysis and cytological features evidence some physiological and structural adaptive traits of the seagrass *Cymodocea nodosa* growing in the vicinity of the Vulcano CO_2_ vents. This adaptation strategy combines the reduction of carbon fixation and gluconeogenesis with increasing photophosphorylation and cell respiration to maintain the high energy demand for leaf expansion and elongation of the mesophyll cell. Our results largely corroborate the findings of previous metabolic and transcriptomic studies carried out in the Vulcano vents, raising additional concerns on the use of volcanic vents as proxies for future acidification conditions. On the other hand, the specificities of the volcanic emissions also raise interesting questions and allow the investigation of pertinent physiological questions.

## Methods

### Sites description and plant sampling

Vulcano, the southernmost island of the Aeolian Archipelago, contains the most recently active center of submersed CO_2_ vents systems^1^. The most recent CO_2_ emissions originated from a volcanic activity on Vulcano occurred in 2002 has caused a series of gas explosions^2^. Most of the active submersed seeps are located along southern and western shores of Baia di Levante, where dispersed underwater leaks cover a 0.13 km^2^ shallow area (1 m depth). Gas composition at the seeps consists of 99% of carbon dioxide and dissolved hydrogen sulphide from the seeps was undetectable at the sampling locations; seawater parameters, daily irradiance and *p*CO_2_ concentration at two sites were reported in Olivé et al.^25^.

For molecular analyses, *C. nodosa* samples (i.e., morphological individuals with two or more shoots) were collected at 5 m depth by SCUBA diving in the acidified site referred as high *p*CO_2_ environment (38°25.057′N-14°57.599′E) and in a nearby site referred as normal *p*CO_2_ environment (38° 25′ 22″ N-14° 57′ 82″ E)^25^. To assure the representation of the seagrass meadows in the study sites, sampling were performed along three grids of 20 × 20 m each with the internal distance between sampled plants of 4–5 m to reach a total sampling of 15 individuals at each site. Once collected, epiphytes on leaf surface were rapidly and carefully removed by a razor, then leaves were rinsed in distilled water and immediately frozen in liquid nitrogen and kept at -80 °C until the protein extraction procedure described in Mazzuca et al.^30^.

For the histological and cytological analyses adult leaves were selected from the 15 individuals at each sampling site, cleaned from the epiphytes, washed in sea water and fixed in 4% formalin in 0.15 M phosphate buffer pH 7.2 and stored refrigerated.

### Extraction and purification of total protein from leaves

Frozen leaves were pooled forming 3 biological replicates, each composed by 5 individuals, because of the low amount of leaf tissue for each shoot. Leaf proteins were extracted by the multistep procedures^28^; for each extraction 1.4 g of pooled leaves were powdered in a mortar in liquid nitrogen until a fine powder was obtained. At this powder a volume of 10% TCA in acetone was added and centrifuged at 13,000 rpm for 5 min at 4 °C. Subsequently, four washes were performed in 80% acetone in water. After centrifugation the pellet containing the precipitated proteins was dried at room temperature. Approximately 0.1 g of powdered tissue was dissolved in 0.8 ml of phenol (buffered with Tris–HCl, pH 8.0, Sigma, St. Louis, MO, USA) and 0.8 ml of SDS buffer (30% sucrose, 2% SDS, 0.1 M Tris–HCl, pH 8., 0,5% 2-mercaptoetanol) in a 2 ml microfuge tube. The samples were vortexed for 30 s and centrifuged at 13,000 rpm for 5 min to allow the solubilization of proteins in the phenol phase. The phenol phase was mixed with five volumes of 0.1 M ammonium acetate in cold methanol, and the mixture was stored at -20 °C for 30 min to precipitate proteins. Proteins were collected by centrifugation at 13,000 rpm for 5 min. Two washes were performed with 0.1 M ammonium acetate in cold methanol, and two with cold 80% acetone, and centrifuged at 13,000 rpm for 7 min. The final pellet containing purified protein was dried and dissolved in Laemmli 1DE separation buffer overnight. Proteins were then quantified by measuring the absorbance at 595 nm according to the Bradford assay. Protein yield was calculated as milligrams of protein for g fresh tissue weight in three biological replicates at each site. For each replicate, two independent extractions were made. The relative abundances of proteins were calculated as mean ± standard error (n = 6). A Student t-test was used to make pair-wise comparisons between normal *p*CO_2_ and high *p*CO_2_ samples. Unless otherwise noted, p-levels of 0.05 were used as the threshold for statistical significance.

### Electrophoresis of leaf proteins, protein in-gel digestion and mass spectrometry analyses

A gel was prepared at a concentration of 10% acrylamide/bisacrylamide, according to the method of^45^. The ratio of acrylamide/bisacrylamide was 12.5% in the running gel and 6% in the stacking gel. All biological replicates were heated for 5 min at 100 °C and 25 μg of activated proteins were loaded on the each well in the gel. The electrophoretic run was carried out at 60 mA for the stacking gel and 120 mA in the running gel at power of 200 V. The electrophoresis ran for an average time of 1 h and 15 min. The gels were stained with Coomassie Blue overnight and subsequently destained with several changes of destaining solution (45% methanol, 10% acetic acid). Digitalized images of the destained SDS-PAGEs were analyzed by the Quantity One 1-D Analysis Software (Bio-Rad Laboratories; Berkeley, California) to measure the optical densities at each lane of all biological replicates from both sites. The amount of protein at bands of 55, 25, and 10 kDa was done using the marker reference bands at 75, 50, and 25 kDa that contained 150, 750, and 750 ng of proteins respectively (Fig. 1). Each lane of the same SDS-PAGE was divided in six slices from 200 to 10 kDa and manually excised from the gel.

The CBB-stained gel slices from three biological replicates were destained and then processed for the reduction and alkylation steps by using dithiotreitol (DTT) and iodoacetamide (IAA), respectively^46^. Gel pieces were digested by Trypsin (Promega, Madison WI, USA) overnight at 37 °C adding ammonium bicarbonate buffer to cover gel matrix. The extracted peptides from three independent biological replicates and two technical replicates were immediately processed for mass spectrometry analysis.

### Tandem mass spectrophotometry (MS) analysis

A data-dependent tandem MS approach was carried out using a hybrid quadrupole-time-of-flight (Q-TOF) mass spectrometer (6550 IFunnel Q-TOF, Agilent Technologies, CA, USA), with a nano LC Chip Cube source (Agilent Technologies, CA, USA) according to Lucini and Bernardo^47^. The chip consisted of a 40-nL enrichment column (Zorbax 300SB-C18, 5 µm pore size) and a 150 mm separation column (Zorbax 300SB-C18, 5 µm pore size) coupled to an Agilent Technologies 1200 series nano/capillary LC system and controlled by the MassHunter Workstation Acquisition (version B.04).

A volume of 8 µL was injected per run, loading peptides onto the trapping column at 4 µL min^−1^ in 2% (v/v) acetonitrile and 0.1% (v/v) formic acid. After enrichment, the chip was switched to separation mode and peptides were back flush eluted into the analytical column, during a 60 min acetonitrile gradient (from 3 to 90% v/v in 0.1% formic acid) at 0.6 µl min^−1^. The mass spectrometer was used in positive ion mode and MS scans were acquired over a mass range from 300 to 1700 m/z, at 4 spectra s^−1^.

Twelve precursor ions per scan were selected for auto-MS/MS, adopting an absolute threshold of 1000 and a relative threshold of 0.01%, and enabling active exclusion after 2 spectra of the same precursor. Ramped collision energy was used for collision-induced decomposition, as a function of peptide charge.

Peptide identification from MS/MS spectra, proteins inference and validation were performed in Spectrum Mill MS Proteomics Workbench (Rev B.04; Agilent Technologies). Auto MS/MS spectra were extracted from raw data accepting a minimum sequence length of 3 amino acids and merging scans with the same precursor within a mass window of ± 0.4 m/z, in a time frame of ± 30 s. Search parameters were Scored Peak Intensity (SPI) ≥ 50%, precursor mass tolerance of ± 10 ppm and product ions mass tolerance of ± 20 ppm. Carbamidomethylation of cysteine was set as fixed modification and trypsin was selected as enzyme for digestion, accepting 2 missed cleavages per peptide.

Considering that a species-specific proteome was not available, the proteome referring to viridiplantae in Uniprot was used; downloaded on April 2015, a total of 144,283 entries can be found according to this criterion.

Auto thresholds were used for peptide identification in Spectrum Mill, to achieve a target 1% false discovery rate. Label-free quantitation, using the protein summed peptide abundance, was carried out after identification.

### Statistical analyses

The results were directly exported to Mass Profiler Professional B.04 (Agilent Technologies) for statistical analysis. Protein intensities were log2 normalized and fold-change analysis was carried out using a threshold of 3. Multivariate Partial Least Square Discriminant Analysis (PLS-DA) was then carried out (N-fold validation, using N = 3 and 10 repeats). The PLS-DA class prediction model loading, i.e. the plot of the weight for each protein in the model within the latent vectors, was used to select those proteins being more discriminant in class prediction (those having a score of above + 0.2 rather than below -0.2). These proteins were exported from the covariance structures in the PLS-DA hyperspace and further discussed.

### Preparation of samples and histological analyses

For each individual, several 5 × 5 mm pieces of six fixed leaves were cutted and washed in 0.15 M phosphate buffer three times for 10 min; subsequently leaf pieces were treated with 1% osmium tetraoxide in phosphate buffer. Leaf pieces were then dehydrated trough the increasing concentration of ethanol solutions. Dehydrate samples were then imbedded in epoxy resin, obtained by mixing Epon 812-Araldite and ethanol (1: 1, v/v) for 4–5 h at 4 °C and then embedded in pure resin overnight at room temperature. The embedded samples were polymerized in an oven at 60 °C for 3 days; then each sample was cut in 0.2 mm thick sections through the ultramicrotome. The sections were transferred onto slides and stained with Methylene Blue. At least ten sections for each leaf sample were observed and photographed at 100 X magnification and digitalized using Image J open source software. A measurement bar of 100 μm as a reference scale was added to the images obtained for subsequent analysis using CellProfiler open source software (Broad Institute; country). The areas of 20 cells per section of the epidermis and parenchyma were measured and the thickness of the epidermis and parenchyma cell wall was also measured and a 100 × 100 μm scale square. The measurements obtained were divided into two datasets of ​​all the samples of the two sites; the first dataset with the comparison of the mean values ​​of the cell area and the second dataset with the comparison of the average values ​​of the wall thickness. Significance of values from both datasets were made by t-student test using the GraphPad Prism 8 software.

## Supplementary information

Supplementary Information 1.

Supplementary Information 2.

Supplementary Information 3.

Supplementary Information 4.
