# Supplementary material for: Leaf proteome modulation and cytological features of seagrass Cymodocea nodosa in response to long-term high CO2 exposure in volcanic vents
Source: Sci Rep. 2020 Dec 18;10:22332. doi: 10.1038/s41598-020-78764-7 (PMC7749125; doi:10.1038/s41598-020-78764-7)
Supplement: Supplementary file 1 — Supplementary Information 1. [file 41598_2020_78764_MOESM1_ESM.docx]

| Biological replicates | Abs_(595nm)_  Mean value | | | Proteins yield  (μg/μl) | Proteins yield  (mg/g  fresh weight) | Proteins yield  (mg/g fresh weight)  mean value* | | |
| --- | --- | --- | --- | --- | --- | --- | --- | --- |
| N*p*CO_2_ -1 | 0.346 | ± | 0.050 | 1.86 | 18.6 | 15.35 | ± | 2.39 |
| N*p*CO_2_ -2 | 0.233 | ± | 0.032 | 1.26 | 12.6 |  |  |  |
| N*p*CO_2_ -3 | 0.276 | ± | 0.009 | 1.49 | 14.9 |  |  |  |
| H*p*CO_2_-1 | 0.155 | ± | 0.002 | 0.83 | 8.3 | 10.74 | ± | 1.82 |
| H*p*CO_2_-2 | 0.250 | ± | 0.003 | 1.35 | 13.5 |  |  |  |
| H*p*CO_2_-3 | 0.105 | ± | 0.005 | 1.04 | 10.4 |  |  |  |

Supplementary Table 1. Spectrophotometrical absorbance (Abs) and proteins yield in adult leaf tissue from *Cymodocea nodosa* living in normal (N) or high (H) *p*CO_2_ environments.

*Values are the mean of three biological replicates and two technical replicates each sample. t-Student, P<0.05
