# Supplementary material for: Leaf proteome modulation and cytological features of seagrass Cymodocea nodosa in response to long-term high CO2 exposure in volcanic vents
Source: Sci Rep. 2020 Dec 18;10:22332. doi: 10.1038/s41598-020-78764-7 (PMC7749125; doi:10.1038/s41598-020-78764-7)
Supplement: Supplementary file 4 — Supplementary Information 4. [file 41598_2020_78764_MOESM4_ESM.docx]

| Supplementary Table 4. Morphological leaf traits, number and area of parenchymatic cells in the mesophyl of plants living in different *p*CO_2_ conditions. | | | | |
| --- | --- | --- | --- | --- |
| Condition | Leaf width* (mm) | Leaf thickness* (mm) | Cells*  (no./mm^2^) | mean cell area*  (μm^2^) |
| H*p*CO^2^ | 3,22 ± 0,52 | 0,22 ± 0,03 | 58 ± 2 | 8000 ± 600 |
| N*p*CO^2^ | 2,76 ± 0,43 | 0,21 ± 0,02 | 96 ± 3 | 5000 ± 300 |
|  |  |  |  |  |

* Values are the mean of measurements performed on tissue sections from six biological replicates for

each conditions (± SE). t-Student, P<0.05
